# Supplementary material for: Trioxolone Methyl, a Novel Cyano Enone-Bearing 18βH-Glycyrrhetinic Acid Derivative, Ameliorates Dextran Sulphate Sodium-Induced Colitis in Mice
Source: Molecules. 2020 May 21;25(10):2406. doi: 10.3390/molecules25102406 (PMC7287650; doi:10.3390/molecules25102406)
Supplement: Supplementary file 1 [file molecules-25-02406-s001.zip › molecules-797510-SM-final/Supplementary File S1 (NMR Spectrum of TM).pdf]

Trioxolone methyl, a novel cyano enone-bearing 18βH-glycyrrhetinic acid derivative, ameliorates dextran sulphate sodium-induced colitis in mice

Andrey V. Markov [1,*,§], Aleksandra V. Sen'kova [1,§], Oksana V. Salomatina [1,2], Evgeniya B. Logashenko [1], Dina V. Korchagina [2], Nariman F. Salakhutdinov [2] and Marina A. Zenkova [1]

[1] Institute of Chemical Biology and Fundamental Medicine, Siberian Branch of the Russian Academy of Sciences, Lavrent'ev ave., 8, 630090 Novosibirsk, Russia; alsenko@mail.ru (A.V.S.), evg_log@niboch.nsc.ru (E.B.L.), marzen@niboch.nsc.ru (M.A.Z.)

[2] N.N. Vorozhtsov Novosibirsk Institute of Organic Chemistry, Siberian Branch of the Russian Academy of Sciences, Lavrent'ev ave., 9, 630090 Novosibirsk, Russia; ana@nioch.nsc.ru (O.V.S.), korchaga@nioch.nsc.ru (D.V.K.), anvar@nioch.nsc.ru (N.F.S.)

* Correspondence: andmrkv@gmail.com (A.V.M.); Tel.: +7-383-363-51-61

Trioxolone methyl [**Methyl 2-cyano-3,12,19-trioxoolean-1(2),9(11),13(18)-trien-30-oate**]: ^{1}H NMR ($CDCl_3$)

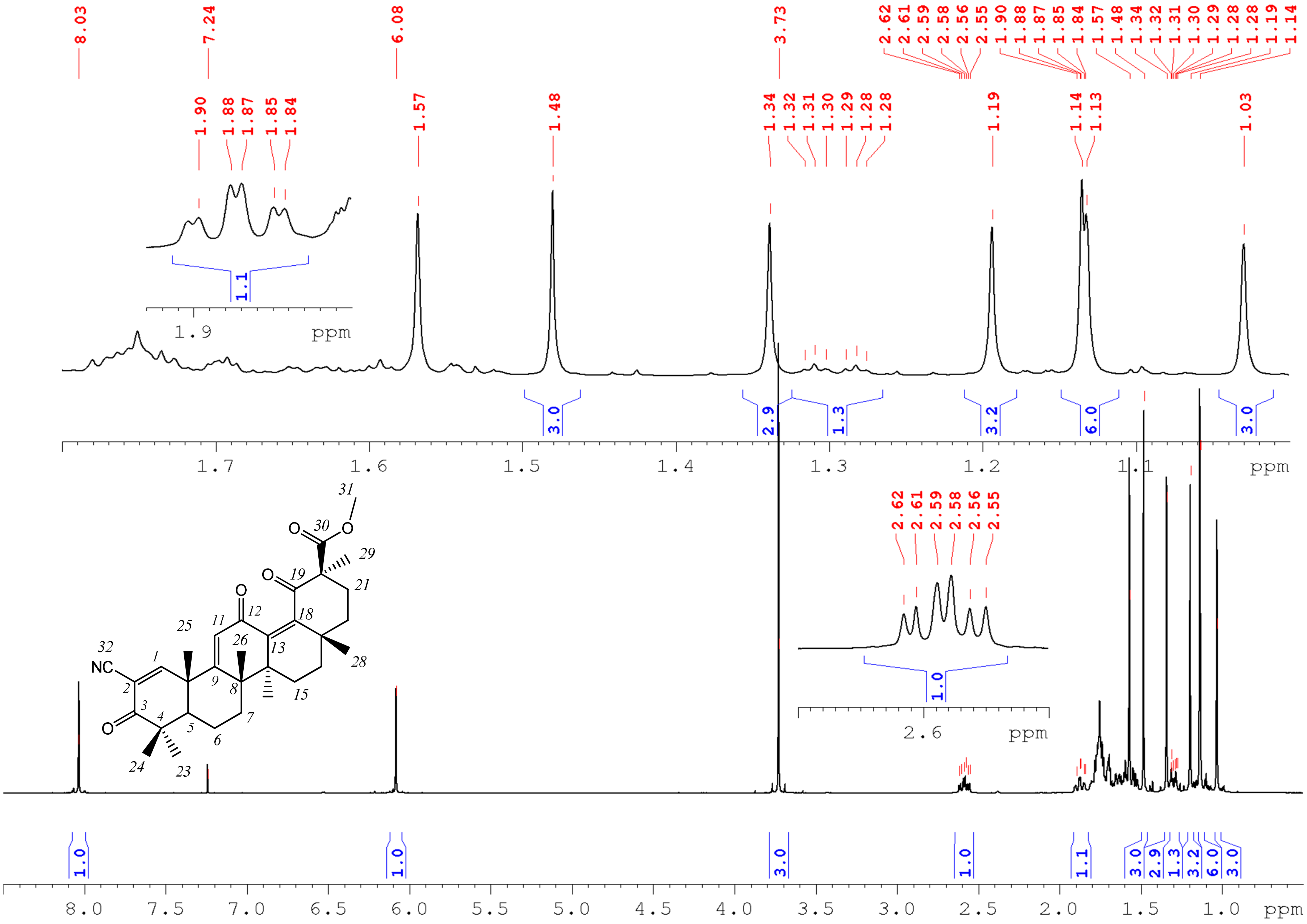

Trioxolone methyl [**Methyl 2-cyano-3,12,19-trioxoolean-1(2),9(11),13(18)-trien-30-oate**]: ^{13}C NMR (J-MOD; 125 MHz) (CDCl$_3$)

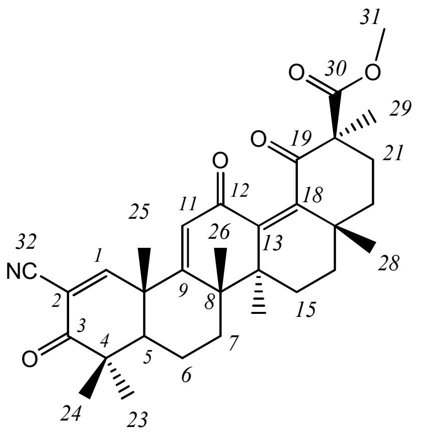

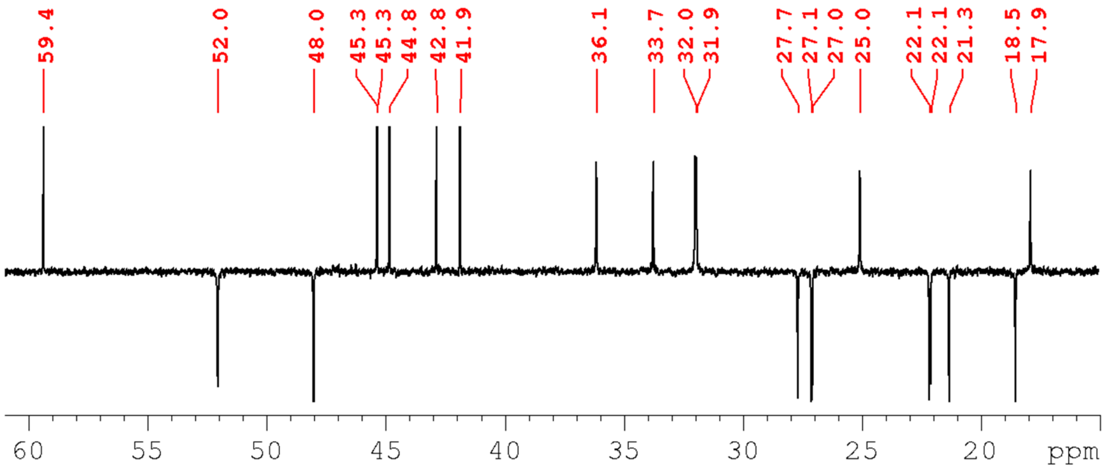

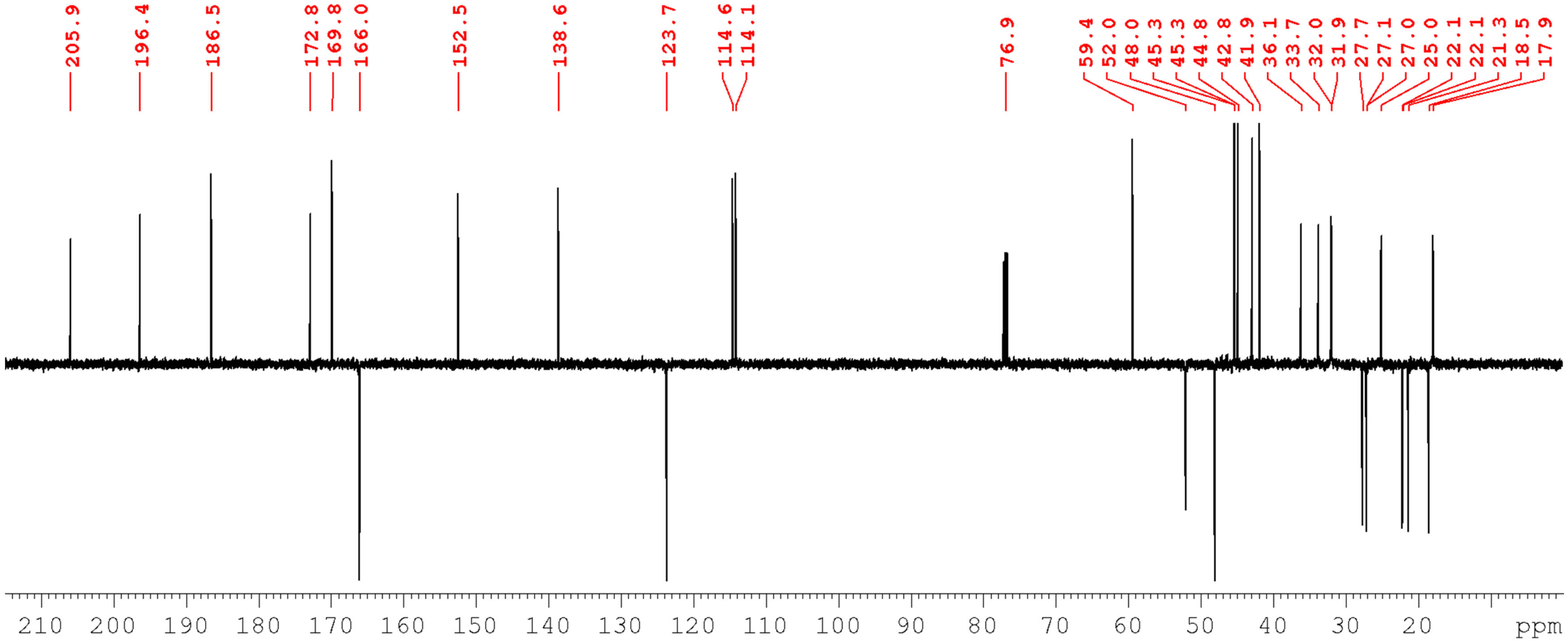

Trioxolone methyl [Methyl 2-cyano-3,12,19-trioxoolean-1(2),9(11),13(18)-trien-30-oate]: IR (KBr)

**Title**	Trioxolone methyl KBr 150:1 Tensor27			**Origin**			
**File Name**				**Date Stamp**	21/4/2020	**Date**	21 Apr 2020 18:00:06
**Technique**	Infrared	**Spectral Region**	IR	**X Axis**	Wavenumber (cm-1)	**Y Axis**	Transmittance
**Spectrum Range**	399.2111 - 3997.8959						
**Points Count**	1867	**Data Spacing**	1.9286				

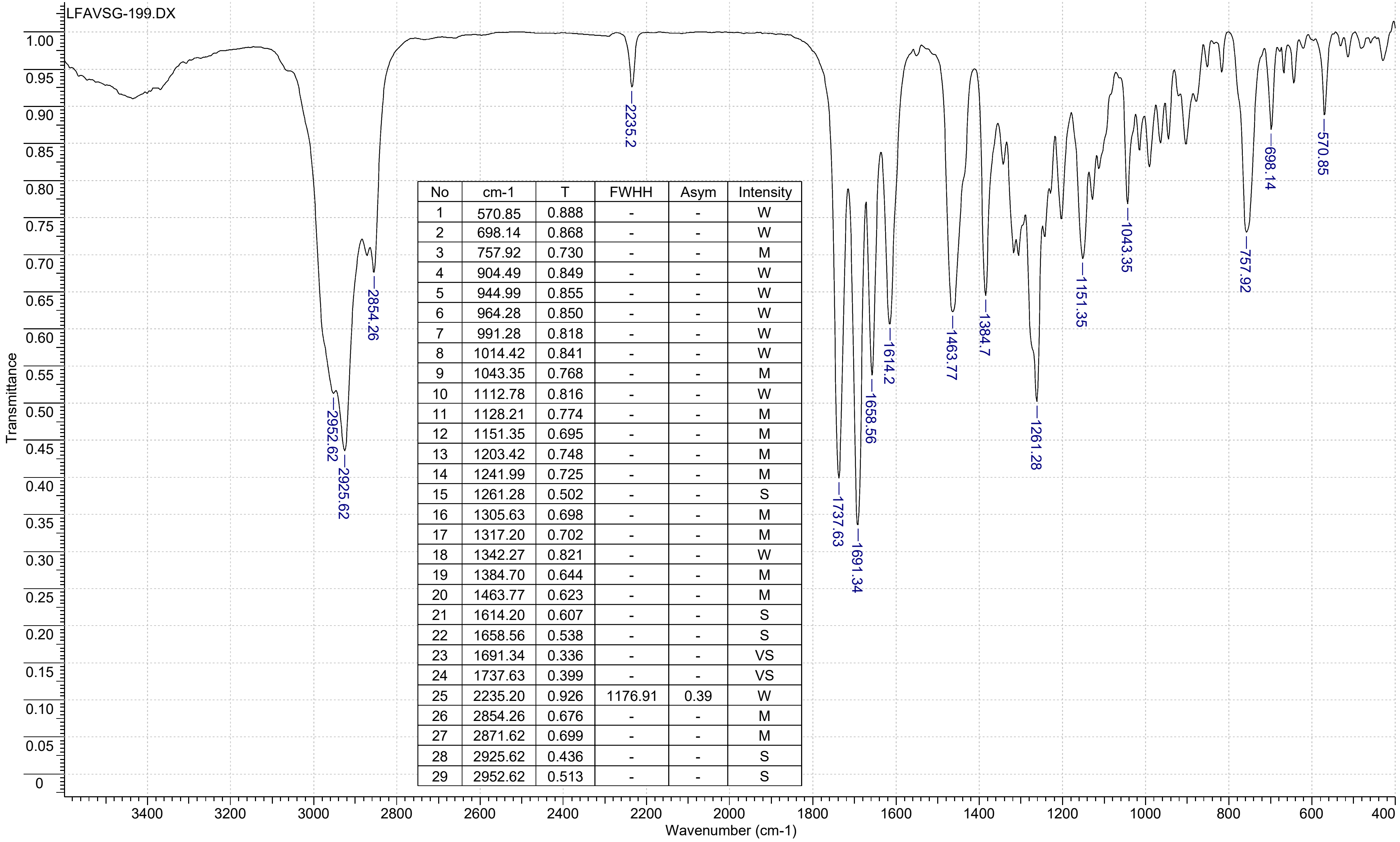

No	cm-1	T	FWHH	Asym	Intensity
1	570.85	0.888	-	-	W
2	698.14	0.868	-	-	W
3	757.92	0.730	-	-	M
4	904.49	0.849	-	-	W
5	944.99	0.855	-	-	W
6	964.28	0.850	-	-	W
7	991.28	0.818	-	-	W
8	1014.42	0.841	-	-	W
9	1043.35	0.768	-	-	M
10	1112.78	0.816	-	-	W
11	1128.21	0.774	-	-	M
12	1151.35	0.695	-	-	M
13	1203.42	0.748	-	-	M
14	1241.99	0.725	-	-	M
15	1261.28	0.502	-	-	S
16	1305.63	0.698	-	-	M
17	1317.20	0.702	-	-	M
18	1342.27	0.821	-	-	W
19	1384.70	0.644	-	-	M
20	1463.77	0.623	-	-	M
21	1614.20	0.607	-	-	S
22	1658.56	0.538	-	-	S
23	1691.34	0.336	-	-	VS
24	1737.63	0.399	-	-	VS
25	2235.20	0.926	1176.91	0.39	W
26	2854.26	0.676	-	-	M
27	2871.62	0.699	-	-	M
28	2925.62	0.436	-	-	S
29	2952.62	0.513	-	-	S
