# Supplementary material for: Trioxolone Methyl, a Novel Cyano Enone-Bearing 18βH-Glycyrrhetinic Acid Derivative, Ameliorates Dextran Sulphate Sodium-Induced Colitis in Mice
Source: Molecules. 2020 May 21;25(10):2406. doi: 10.3390/molecules25102406 (PMC7287650; doi:10.3390/molecules25102406)
Supplement: Supplementary file 1 [file molecules-25-02406-s001.zip › molecules-797510-SM-final/Supplementary Table S1 (probable targets of TM).pdf]

Trioxolone methyl, a novel cyano enone-bearing 18βH-glycyrrhetinic acid derivative, ameliorates dextran sulphate sodium-induced colitis in mice

Andrey V. Markov [1,*,§], Aleksandra V. Sen'kova [1,§], Oksana V. Salomatina [1,2], Evgeniya B. Logashenko [1], Dina V. Korchagina [2], Nariman F. Salakhutdinov [2] and Marina A. Zenkova [1]

[1] Institute of Chemical Biology and Fundamental Medicine, Siberian Branch of the Russian Academy of Sciences, Lavrent'ev ave., 8, 630090 Novosibirsk, Russia; alsenko@mail.ru (A.V.S.), evg_log@niboch.nsc.ru (E.B.L.), marzen@niboch.nsc.ru (M.A.Z.)

[2] N.N. Vorozhtsov Novosibirsk Institute of Organic Chemistry, Siberian Branch of the Russian Academy of Sciences, Lavrent'ev ave., 9, 630090 Novosibirsk, Russia; ana@nioch.nsc.ru (O.V.S.), korchaga@nioch.nsc.ru (D.V.K.), anvar@nioch.nsc.ru (N.F.S.)

* Correspondence: andmrkv@gmail.com (A.V.M.); Tel.: +7-383-363-51-61

§ These authors contributed equally to this work

Supplementary Table S1: The list of predicted primary protein targets of TM.

Target prediction tool	Target ID	Target name
PPB2	Hsd11b1	Hydroxysteroid 11-Beta Dehydrogenase 1
	Kcnh2	Potassium Voltage-Gated Channel Subfamily H Member 2
	Nr3c1	Glucocorticoid Receptor
	Oprm1	Opioid Receptor Mu 1
	Kdr	Kinase Insert Domain Receptor
	Slc6a4	Sodium-Dependent Serotonin Transporter
	Gpr119	G Protein-Coupled Receptor 119
	Hsd11b2	Hydroxysteroid 11-Beta Dehydrogenase 2
	Gsk3b	Glycogen Synthase Kinase 3 Beta
	Chrm3	Muscarinic Acetylcholine Receptor M3
	Cyp19a1	Cytochrome P450 Family 19 Subfamily A Member 1
	Oprd1	Opioid Receptor Delta 1
	Cnr1	Cannabinoid Receptor 1
	Oprk1	Opioid Receptor Kappa 1
	Tnf	Tumor Necrosis Factor-Alpha
	Drd2	Dopamine Receptor D2
PPB2, STITCH	Nos2	Inducible NO Synthase
STITCH	Nos1	Constitutive NO Synthase
	Src	Proto-Oncogene Tyrosine-Protein Kinase Src
	Myc	MYC Proto-Oncogene, BHLH Transcription Factor
	Akt1	AKT Serine/Threonine Kinase 1
	Stat3	Signal Transducer And Activator Of Transcription 3
	Mapk8	Mitogen-Activated Protein Kinase 8
	Hmox1	Heme Oxygenase 1
	Tert	Telomerase Reverse Transcriptase
	Dnmt1	DNA Methyltransferase 1
